# *QuickStats:* Age-Adjusted Percentage*^,^^†^ of Adults Aged ≥65 Years Who Had an Influenza Vaccine in the Past 12 Months,^§^ by Poverty Status^¶^ — National Health Interview Survey, United States, 1999–2001 and 2014–2016

**DOI:** 10.15585/mmwr.mm6707a8

**Published:** 2018-02-23

**Figure:**
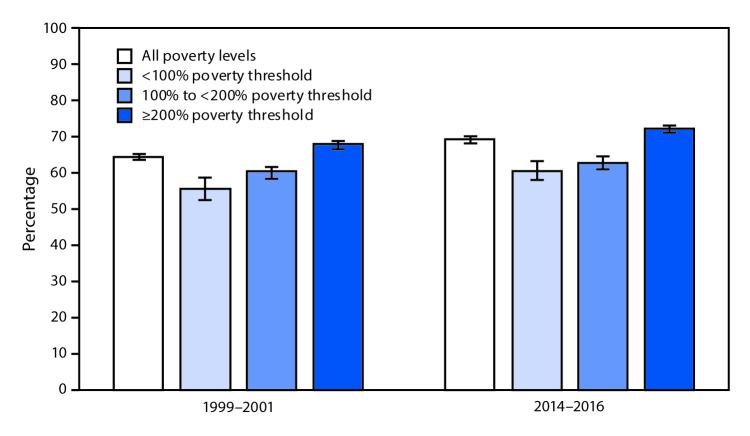
During 2014–2016, 69.2% of all older adults, aged ≥65 years, had received an influenza vaccine in the past 12 months. The percentage of older adults with family income ≥200% poverty level who had received an influenza vaccine in the past 12 months significantly increased from 67.9% during 1999–2001 to 72.2% during 2014–2016. During the same period, the changes from 55.7% to 60.8% among those at the <100% poverty level and from 60.3% to 62.9% for those at the 100% to <200% poverty level were not statistically significant. During both periods, older adults with income ≥200% poverty level were significantly more likely to receive an influenza vaccine compared with those with lower family income.

**Source:** National Health Interview Survey, 1999–2016. https://www.cdc.gov/nchs/nhis.htm.

